# Effect of Music Listening on Physiological Condition, Mental Workload, and Driving Performance with Consideration of Driver Temperament

**DOI:** 10.3390/ijerph16152766

**Published:** 2019-08-02

**Authors:** Huiying Wen, N. N. Sze, Qiang Zeng, Sangen Hu

**Affiliations:** 1 School of Civil Engineering and Transportation, South China University of Technology, Guangzhou 510641, China; 2 Jiangsu Province Collaborative Innovation Center of Modern Urban Traffic Technologies, Southeast University Road #2, Nanjing 211189, China; 3 Department of Civil and Environmental Engineering, The Hong Kong Polytechnic University, Hong Kong, China; 4 Department of Electrical Engineering, The Hong Kong Polytechnic University, Hong Kong, China; 5 School of Civil and Transportation Engineering, Guangdong University of Technology, Guangzhou 510006, China

**Keywords:** in-vehicle music, music genre, driver temperament, physiological condition, driving performance

## Abstract

This paper presents the study on the association between in-vehicle music listening, physiological and psychological response, and driving performance, using the driving simulator approach, with which personality (temperament) was considered. The performance indicators considered were the standard deviation of speed, lane crossing frequency, perceived mental workload, and mean and variability of heart rate. Additionally, effects of the presence of music and music genre (light music versus rock music) were considered. Twenty participants of different personalities (in particular five, four, seven, and four being choleric, sanguine, phlegmatic, and melancholic, respectively) completed a total of 60 driving simulator tests. Results of mixed analysis of variance (M-ANOVA) indicated that the effects of music genre and driver character on driving performance were significant. The arousal level perceived mental workload, standard deviation of speed, and frequency of lane crossing were higher when driving under the influence of rock music than that when driving under the influence of light music or an absence of music. Additionally, phlegmatic drivers generally had lower arousal levels and choleric drivers had a greater mental workload and were more likely distracted by music listening. Such findings should imply the development of cost-effective driver education, training, and management measures that could mitigate driver distraction. Therefore, the safety awareness and safety performance of drivers could be enhanced.

## 1. Introduction

Music listening is a popular in-vehicle activity, especially of drivers, round the world [1,2,3]. In the recent decade, automobiles have been the most popular location, among others, for music listening [4]. In-vehicle music listening can induce not only entertainment but also as a stimulus to drivers. This can in turn help mitigate the boredoms and drowsiness of drivers (conceptualized as ‘self-therapy’) [2]. For instance, Stutts et al. found that drivers were more likely to listen to music when driving alone, under dark conditions, and moderate to heavy traffic conditions [5]. However, to the best of our knowledge, it was rare that the ‘self-therapy’ effect of music listening on driving performance was attempted. In particular, effects of music type, sound level, and other possible confounding factors on the physiological performances of drivers in terms of arousal, emotion, and mental workload and therefore driving performance should be investigated [1,3,6,7]. Additionally, the interaction between music, physiological factors, and the driving performance of drivers can moderate crash risk [4,8]. 

Effects of music listening on driving performance can generally be classified into two types: (i) Distraction and (ii) arousal [2]. Distraction refers to the shift of the driver’s attention away from the driving task, which is a complex process requiring a high level of cognitive, sensory, and locomotor skills. Being a competing stimulus to the driver, in-vehicle music listening could increase the driver’s mental workload, and thus impair the driving performance. Indeed, driving and music listening both compete for the limited cognitive capability of the driver [6]. As revealed in a study by the National Highway Traffic Safety Administration of the United States [9], 25% of road crashes were attributed to in-vehicle driver distraction, including music-listening, having a conversation with passengers, etc. Additionally, under adverse environmental and traffic conditions, such as hilly terrain and low visibility conditions [10,11], music listening might remarkably increase the driver’s mental workload and driving impairment could be magnified [12]. Regarding arousal, it refers to the level of alertness of the driver. Arousal levels can range from asleep to highly energetic. In-vehicle music listening could possibly increase the degree of arousal of a driver. Not only the driving performance, but also the driver’s physiological performance can be improved by music listening. In particular, music listening during driving was effective in relieving stress, calming down emotions, and avoiding drowsiness of the driver [13]. Cummings et al. found that the risk of crashing when driving and music listening was lower than driving with an absence of music listening [14]. However, the extent of crash risk reduction might vary with the driving scenario and traffic condition [15,16]. 

The anticipated (both negative and positive) impacts of music listening on driving performance can be moderated by possible confounding factors, including driver demographic and socio-economic characteristics (e.g., age, gender, education level, and temperament), music type (characterized by tempo, intensity, emotion, and genre), traffic flow condition, geometric design, and road environments [1,2,3,17,18]. Therefore, variation in the anticipated changes in driving performance is considerable, and the effect of music listening on driving performance may be controversial. For example, van der Zwaag et al. indicated that faster tempo music was correlated to a higher level of arousal [19], but Brodsky suggested that no evidence could be established for a significant association between the level of arousal and music tempo [1]. 

Dibben and Williamson suggested that music genre could possibly affect driving performance [2], though the association between music genre and driving performance was rarely measured. A number of attributes, including sound intensity and tempo, can characterize music type or genre [1,17]. For example, light music usually has a slow tempo while rock music often has a high sound intensity. Additionally, compared to tempo and other sound attributes, music genre may be more understandable to the non-professional. Therefore, it would be more efficient to measure the association between the genre of music, psychological performance, and driving performance for the development of effective road safety education and promotion strategies.

Regarding driver characteristics, an examination of the modification effects by driver demographics and socio-economics on the association between music listening, emotion, and driving performance is rare [1,2]. Indeed, drivers’ characters, also known as temperament, could moderate the impaired driving performance while listening to music [20]. There are four major temperament types: (i) Choleric, (ii) sanguine, (iii) phlegmatic, and (iv) melancholic [21]. In particular, choleric drivers tend to be more aggressive and have a higher propensity to commit traffic offenses, such as speeding and unsafe overtaking. Response times of sanguine drivers in an emergency are low. However, sanguine drivers tend to be less patient especially under monotonic road environment and traffic conditions, and sanguine drivers are more likely being distracted by other secondary tasks (e.g., talking to passengers). Phlegmatic drivers tend to be more obedient to the traffic regulations but have longer reaction times in an emergency. In contrast, melancholic drivers tend to be risk-averse [20,22]. It is therefore believed that the heterogeneity effect influences the association between music listening, arousal, emotion, and driving performance and distraction because of the intervention by driver temperament.

A few studies have attempted to investigate the safety issue attributed to driver distraction by music listening using one of two conventional approaches: (i) Diagnosis of empirical crash data and observational survey; and (ii) simulated driving experiments in the laboratory and on-road tests [2]. The former is capable of examining the safety impacts of the prevalence of music listening but not the mechanism between music listening, emotion, and actual driving performance. In contrast, the latter was capable of measuring the psychological and driving performance directly [1,6,7,17,19]. Therefore, factors, including music genre, road environments, and traffic conditions, contributing to psychological and driving performance and their interactions can be revealed. For the simulated driving experiment, possible driving performance indicators are speed, lateral position, response time, and frequency of traffic violation [6,17]. For the physiological performance, possible indicators that are correlated to arousal level, emotion, and mental workload are heart rate and respiration rate [1,3,6,7]. Additionally, it is possible to assess the perceived performance of drivers using a self-reported survey after the driving simulator experiment.

The purpose of this study is to examine the interactions between in-vehicle music listening and physiological and driving performance, taking into account the effects of music genre and drivers’ temperament. In this study, 20 participants completed three driving simulator trails. The driving performance indicators considered were standard deviation of speed and lane-crossing frequency. Additionally, physiological performance in terms of the mean and variability of heart rate was measured. Furthermore, self-reported mental workload was considered. Then, analysis of variance (ANOVA) was conducted to evaluate the differences in physiological and driving performance between music genres and drivers’ temperaments. Results should be indicative of the enforcement and driver education measures that could enhance road safety. 

## 2. Study Design 

### 2.1. Participants

Twenty participants completed the driving simulator experiments. They were university students from the South China University of Technology. The inclusion criteria were holding a valid driving license and in good health condition, e.g., with no symptom of simulator sickness and visual/audio impairment. Of the 20 participants, 12 were male. The mean age was 25 (ranged from 22 to 27 years) and average driving experience was three years. Prior to the driving simulator experiment, each participant was invited to complete an aptitude test, namely the ‘Temperament Testing Questionnaire’. The questionnaire was first developed by Huichang Chen from Beijing Normal University [23]. It is a standard assessment tool of temperament for the Chinese population. In the questionnaire, there are 60 assessment items. The 60 items can be categorized into four groups, each of which is specified for one particular temperament type, e.g., choleric, sanguine, phlegmatic, and melancholic, etc. For every assessment item, a 5-point scale approach was used to gauge the participant’s perception. In particular, a score was given to indicate the degree of matching between the scenario described and their own situation, for example, −2 refers to totally mismatched; −1 refers to relatively mismatched; 0 refers to neutral; 1 refers to relatively matched; and 2 refers to totally matched. For each of the four temperament groups, the overall score was estimated. The score was then used to determine the personality of the participant. According to the results of the questionnaire survey, the number of participants classified as choleric, sanguine, phlegmatic, and melancholic was 5, 4, 7, and 4 respectively.

### 2.2. Music Genre

In this study, two music genres, (i) rock and (ii) light music, were considered. These are the two most popular music genres among Chinese drivers [24]. For each music genre, 10 music tracks, from a popular Chinese online music archive—NetEase, were chosen. For the rock music, tempos of the soundtracks were all above 120 bpm; for the light music, tempos were all below 80 bpm. In the simulated driving experiments, music was played via a music player software and exposed to participants by the speaker of a nearby laptop. Participants were free to adjust the sound volume and select his or her preferred soundtracks during the experiment. This was to enhance the ecological validity of the experiments.

### 2.3. Driving Simulator Apparatus and Driving Scenario

**Figure 1:**
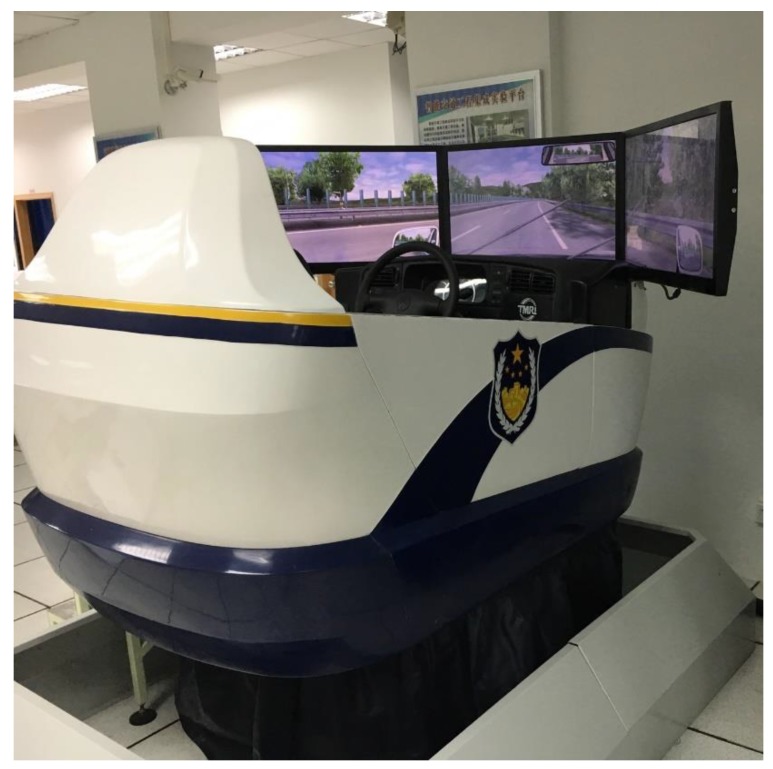
Fix-based driving simulator in the South China University of Technology.

The simulated driving experiments were carried out using a fix-based driving simulator in the South China University of Technology (as shown in Figure 1). The system could provide a 210° horizontal view. Sensors were installed in the steering wheel, accelerator, and brake pedal to capture the information on driving performance, including the steering angle, acceleration, and braking time and force. For the physiological performance, a mobile and mountable sensor—BIOPAC^TM^ MP36R 4-Channel System (UPWARDS TEKSYSTEMS, Ltd., Beijing, China)—was applied to record the metrics, including heart rate, at a frequency of 10,000 Hz.

For the road environment, the simulated road was a two carriageway six-lane suburban highway. The driving scenario was in the daytime, with good lighting and fine weather conditions. The traffic flow condition was moderate, and the posted speed limit was 60 km/h. This was to simulate the road environment of a typical suburban highway in China. 

### 2.4. Experimental Procedure

Before the experiments, we informed the participants the purpose, requirements, and possible physiological impacts of the experiments and briefly described the simulated driving scenes. Then, they filled in the basic information form and the commitment letter. Each participant was invited to complete three simulated driving trials, each for 20 min. In each of the driving simulator trials, the participant was exposed to one of three music conditions: (i) Rock music; (ii) light music; and (iii) no music (set as ‘control’). A 10-min break was given between two successive trials, and the order of the music conditions was counterbalanced among the 20 participants. Prior to the experiment, a 20-min practice session was given to every participant to help one familiarize with the operation of the driving simulator and the simulated driving scenario. After each simulated driving trial, the participant was also asked to complete a questionnaire—NASA Task Load Index (NASA-TLX). NASA-TLX is a well-established and widely adopted assessment tool for perceived mental workload and emotion from six perspectives: Mental demand, physical demand, temporal demand, performance, effort, and frustration. The drivers rated on six subscales for each simulated driving trial within a 100-points range with 5-point steps. The scores on the six subscales were summed as a NASA-TLX score. A high NASA-TLX score implies a high mental workload. 

## 3. Method of Analysis

In this study, the driving performance indicators considered were the standard deviation of speed and lane crossing frequency in each trial. In particular, the standard deviation of speed, $\sigma_{v}$, can be computed using the following expression:

(1) $$\sigma_{v}=\sqrt{\frac{1}{N}\sum_{i=1}^{N}{\left(v_{i}-\bar{v}\right)}^{2}},$$

where $v_{i}$ refers to the instantaneous speed of the ith observation, *N* refers to the total number of observations in a trial, and $\bar{v}$ refers to the average speed of a trial.

For the physiological performance, the indicators used were the mean and variability of the heart rate of a trial. In this study, the cardiovascular activities of participants were recorded using an electrocardiogram (ECG) by the BIOPAC^TM^ MP36R 4-Channel System and pre-processed by the Acq*Knowledge* software [25]. The software automatically detected the R-peaks in the ECG, after the amplification and filtering processes. The mean heart rate was calculated as the number of R-peaks within one minute. The low frequency and high frequency of R-peaks were obtained via Fourier transformation. The ratio of high frequency to low frequency, LF/HF, was used to represent the variability of heart rate. 

To evaluate the difference in the driving performance and physiological performance between trials (of different driver characteristics and music genre), mixed-analysis of variance (M-ANOVA) was applied. In particular, M-ANOVA can assess both the between-subject (driver temperament) and within-subject (music genre) effects on driving performance. The outcome variables were the mean and variability of heart rate, NASA-TLX score, standard deviation of speed, and lane crossing frequency. The effect size of a possible risk factor was indicated using the Partial eta square ($\eta_{p}^{2}$, at the 5% level of statistical significance, two-tailed). The proposed M-ANOVA was conducted using the statistical analysis package SPSS [26].

## 4. Results

### 4.1. Physiological State

#### 4.1.1. Mean Heart Rate

**Figure 2:**
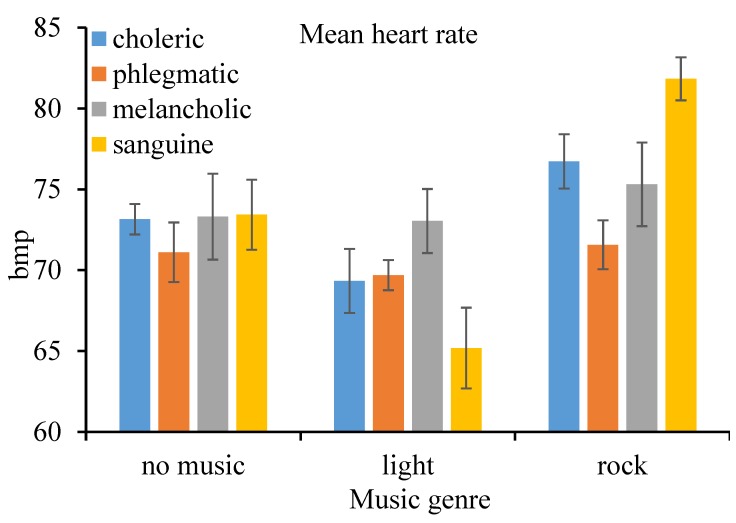
Mean heart rate (and standard error) by music genre and personality.

The results of M-ANOVA are summarized in Table 1. According to the results, the within-subject—music genre (*F*(2, 32) = 88.44, *p* < 0.001, $\eta_{p}^{2}$ = 0.847), between-subject effect—driver temperament (*F*(3, 16) = 7.109, *p* = 0.003, $\eta_{p}^{2}$ = 0.571), and their interaction effects (*F*(6, 32) = 20.08, *p* < 0.001, $\eta_{p}^{2}$ = 0.790) on the mean heart rate were all statistically significant at the 1% level. Results of pairwise comparisons indicated that the mean heart rate when driving under the influence of rock music was higher than that driving under the influence of no music. In contrast, the mean heart rate when driving under the influence of light music was lower than the control, both at the 1% level of significance. Additionally, driver temperament also moderated physiological performance. In particular, the mean heart rate of phlegmatic drivers was significantly lower than that of the counterparts, all at the 5% level (choleric: *p* = 0.041, melancholic: *p* = 0.007, sanguine: *p* = 0.021). Nevertheless, sanguine drivers presented lower mean heart rates (mean = 65.179, standard deviation = 2.500) under light music conditions while higher mean heart rates (mean = 81.827, standard deviation = 1.332) under rock music conditions, compared to the drivers of other groups, as shown in Figure 2.

#### 4.1.2. Heart Rate Variability

**Figure 3:**
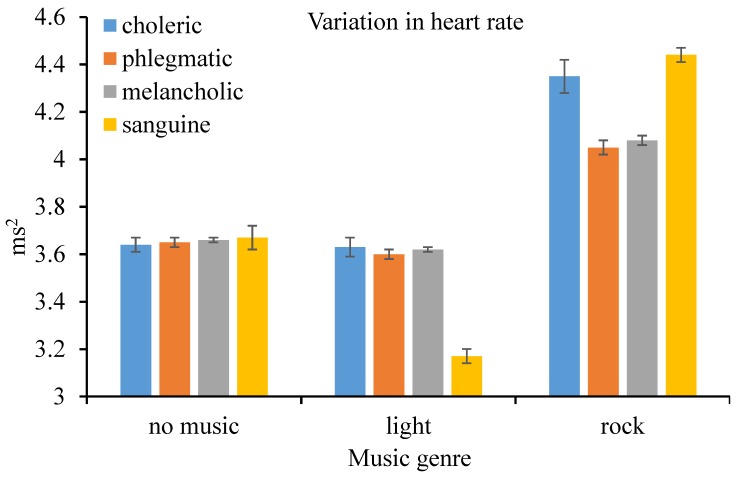
Variation in heart rate (and standard error) by music genre and personality.

According to the results of M-ANOVA, the within-subject effect–music genre (*F*(2, 32) = 3108, *p* < 0.001, $\eta_{p}^{2}$ = 0.995), between-subject effect–driver temperament (*F*(3, 16) = 14.25, *p* < 0.001, $\eta_{p}^{2}$ = 0.728), and their interaction (*F*(6, 32) = 170.2, *p* < 0.001, $\eta_{p}^{2}$ = 0.970) on the variability of heart rate were all found to be significant at the 1% level. Results of pairwise comparison indicated that variability of the heart rate when driving under the influence of rock music was higher than that without music; again, variability of the heart rate when driving under the influence of light music was lower than that without music, both at the 1% level of significance. Furthermore, variability of the heart rate of choleric drivers was again higher than that of the counterparts. Nevertheless, variability of the heart rate of sanguine drivers was sensitive to the music genre. For instance, the variability was particularly high when driving under the influence of rock music but very low when driving under the influence of light music (as shown in Figure 3).

### 4.2. Perceived Mental Workload

**Figure 4:**
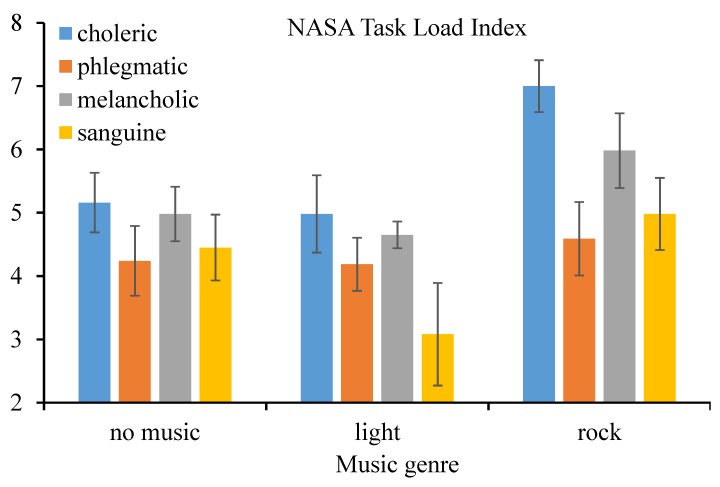
Perceived mental workload (and standard error) by music genre and personality.

For the perceived mental workload, in accordance to the results of M-ANOVA, the within-subject effect–music genre (*F*(2, 32) = 211.7, *p* < 0.001, $\eta_{p}^{2}$ = 0.930) on the NASA-TLX score was statistically significant at the 1% level. In particular, the perceived mental workload when driving under the influence of rock music was higher, and that when driving under the influence of light music was lower, compared to that without music listening. Additionally, the between-subject effect–driver temperament (*F*(3, 16) = 10.0, *p* = 0.001, $\eta_{p}^{2}$ = 0.652) and interaction effect (*F*(6, 32) = 29.33, *p* < 0.001, $\eta_{p}^{2}$ = 0.846) on perceived mental workload were both significant at the 1% level. Consistently, the perceived mental workload of choleric drivers was higher than that of the counterparts (as shown in Figure 4). 

### 4.3. Driving Performance

**Figure 5:**
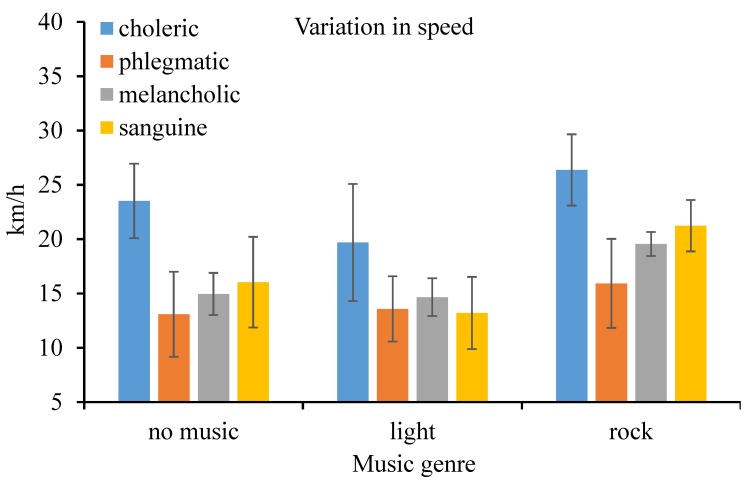
Variation in speed (and standard error) by music genre and personality.

For driving performance, the standard deviation of speed and lane crossing frequency were assessed. In particular, the within-subject (music genre), between-subject (driver temperament), and their interaction on driving performance were investigated. According to the results of M-ANOVA, both the within-subject (*F*(2, 32) = 30.05, *p* < 0.001, $\eta_{p}^{2}$ = 0.653) and between-subject effect (*F*(3, 16) = 9.495, *p* = 0.001, $\eta_{p}^{2}$ = 0.640) on the standard deviation of speed were significant at the 1% level. The interaction effect might affect the standard derivation of speed, however, no significant association could be revealed (*F*(6, 32) = 1.444, *p* = 0.229, $\eta_{p}^{2}$ = 0.213). Standard deviation of speed when driving under the influence of rock music was higher than that under light music and an absence of music. Additionally, the standard deviation of speed of choleric drivers was higher than that of the counterparts, regardless of the prevalence of music and music genre (as shown in Figure 5).

**Figure 6:**
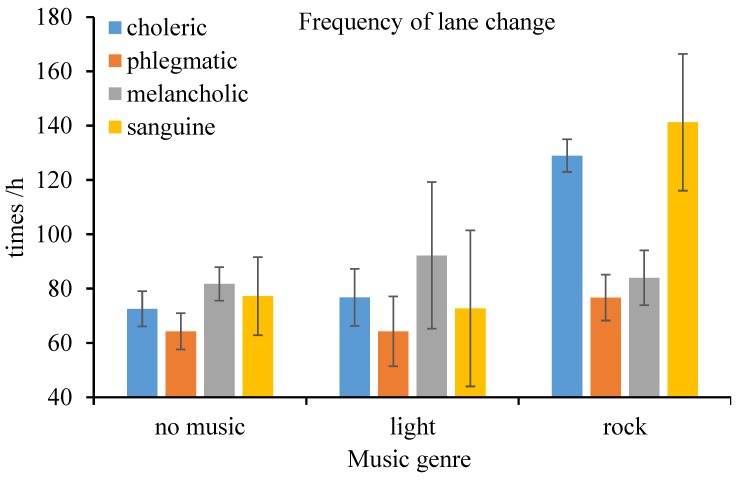
Frequency of lane change (and standard error) by music genre and personality.

According to the results of M-ANOVA, the within-subject effect–music genre (*F*(2, 32) = 47.88, *p* < 0.001, $\eta_{p}^{2}$ = 0.750), between-subject effect–driver temperament (*F*(3, 16) = 8.021, *p* = 0.002, $\eta_{p}^{2}$ = 0.601), and their interaction effect (*F*(6, 32) = 11.97, *p* < 0.001, $\eta_{p}^{2}$ = 0.692) on the frequency of lane crossing were statistically significant, all at the 1% level. Again, frequency of lane crossing when driving under the influence of rock music was higher than that under the influence of light music and absence of music. Furthermore, the lane crossing frequency of phlegmatic drivers was less than that of choleric and sanguine drivers (as shown in Figure 6).

## 5. Discussion

Music listening has long been a popular in-vehicle activity. In particular, it can mitigate boredom when driving alone [2]. However, an investigation of the effect of music genre on physiological, mental, and driving performance is rare. Additionally, the intervention effect of the driver’s personal character on the association between music type and driving performance should be explored. In this study, effects of music genre (e.g., rock and light music) and driver temperament on physiological and driving performance were evaluated using the driving simulator approach. Results of M-ANOVA indicated that both the within-subject effect (music genre) and between-subject effect (driver temperament) on the mean and variability of heart rate, frequency of lane crossing, and standard deviation of speed were significant. Additionally, the interaction effect between music genre and driver temperament was prevalent. Such findings should be indicative of the driver education and promotion strategies that could in turn enhance driving safety.

### 5.1. Effects of Music Genre

For physiological performance, the mean and variability of heart rate when driving under the influence of rock music were both higher than that under the influence of light music and an absence of music. This implies that the driver’s arousal level could increase when driving under the influence of rock music [1,6]. This is consistent with the findings of previous studies [2,19]. In particular, rock music is often characterized as having a fast tempo and high sound volume. Additionally, some complicated rhythmic patterns and layered textures could be prevalent in rock music. Therefore, drivers could be easily motivated and have a higher arousal level when driving under the influence of rock music [2]. On the other hand, both the perceived and revealed mental workload of drivers could be increased when driving under the influence of rock music, because of the increase in the demand for information processing [6]. Such findings are indeed consistent with the analysis results of the NASA-TLX score in the self-report survey. With respect to driving performance, the standard deviation of speed and frequency of lane crossing were both higher when driving under the influence of rock music, which indicated a higher crash risk [27,28,29,30]. These were again consistent with the findings of previous studies [1,6]. Indeed, an increase in mental workload demands while driving under the influence of rock music could impair driving performance. This could in turn alleviate the negative impact on the emotion of drivers [16]. 

In contrast, light music can be categorized as slow and relatively quiet. Light music can indeed calm down a person [2]. The relieving effect of light music could possibly reduce the arousal level and mental workload of drivers. Therefore, the mean and variability of the heart rate and NASA-TLX score were lower while driving under the influence of light music, as revealed in the current driving simulator study. Even though light music could remarkably enhance drivers’ physiological (heart rate) and psychological (perceived workload) performance, its effects on driving performance (as indicated by the standard deviation of speed and frequency of lane crossing) were marginal. This could be attributed to the conflicting effect of distraction by light music on driving performance. For instance, the relationship between distraction by light music and driving impairment could be negligible [6], and just the level of alertness of the driver could be reduced when one was relaxed (i.e., having a negative impact on driving performance) [2]. In other words, the effect of driving under the influence of light music could be comparable to that when music was absent.

### 5.2. Effects of Driver Temperament

Results of the driving simulator study indicated that the mean heart rate of phlegmatic drivers was lower than that of other driver groups, regardless of the presence of music and music genre. This indicated that phlegmatic drivers have a lower arousal level in general [1,6]. This is expected as phlegmatic people can be categorized as calm and humble. A phlegmatic person is unlikely to be motivated by any stimulus [21]. Additionally, a phlegmatic person has a higher tolerance to relatively simple and monotonous tasks. Therefore, the level of arousal of phlegmatic drivers was lower in general. This could be reflected by more risk-averse driving behavior (i.e., lower frequency of lane crossing [20]). 

With respect to the perceived mental workload (NATA-TLX score) and revealed physiological performance (heart rate), driving impairment of choleric drivers was more remarkable (both higher variability of heart rate and NASA-TLX score) than the counterparts, regardless of the presence of music and music genre. Such findings are not surprising since choleric persons are usually enthusiastic. They rapidly respond to any external stimulus and challenging tasks [21]. Indeed, external stimulus and challenging tasks induce a higher demand for cognition and information processing, and the mental workload is therefore increased. For the revealed driving performance, the standard deviation of speed of choleric drivers was also higher than that of the counterparts. This was consistent with the findings of previous studies indicating that choleric drivers tend to drive more aggressively [22]. 

For sanguine drivers, the interaction by music genre on the association between physiological performance and driver character was noticeable. In particular, the mean and variability of heart rate when driving under the influence of rock music were remarkably higher than that of light music (See Figure 1 and Figure 2). Both the arousal level and mental effort of sanguine drivers were higher when driving under the influence of rock music. This was also expected since sanguine drivers tended to have a good adaption capability, and they were more prone to driving distraction [21]. Furthermore, both the perceived mental workload and lane crossing frequency of sanguine drivers was low when driving under the influence of rock music (See Figure 5). 

Lastly, for melancholic drivers, no evidence could be established for the association between physiological performance, driving behavior, and personality. Indeed, the driving performance of melancholic drivers was comparable to that of other driver classes, regardless of the music genre. This is consistent with the findings of a previous study [22].

The above findings are useful for the development of effective driver education strategies, in particular, a promotional and publicity program that could enhance public awareness of the (negative) impact of music listening on driving performance. To the best of our knowledge, it is the first time that personality, in terms of driver temperament, was considered for the association between music genre, physiological, and driving performance. This should be indicative for transport operators for the development of cost-effective and sustainable driver management measures that could mitigate the crash risk attributed to driving under the influence of music.

## 6. Conclusions

This paper presents a study on the effect of music listening on physiological and psychological performance, and therefore the driving performance using the driving simulator approach. The interaction effect of the driver temperament (character) on the association was also considered. The results indicated that driving under the influence of rock music was correlated to higher arousal levels and perceived mental workload, and that of light music was marginally correlated to driving impairment. For driver personality, the performance of phlegmatic drivers was found to be less sensitive to music genre, however, that of choleric drivers was sensitive to music genre. Such findings are insightful for the development of comprehensive driver education, training, and management measures, especially for transport operators, that could mitigate the risk of driver distraction.

Nonetheless, there are some limitations. First, only two music genres were considered. Indeed, the tempo and sound intensity of the music could also affect the mental workload, physiological, and driving performance. It would be worth exploring the effect size of tempo and sound intensity of music on the physiological, psychological, and driving performance, if more comprehensive information on sound attributes are available. On the other hand, due to the limited budget, only 20 participants were recruited in this study and they were all students from the same university. It is necessary to investigate the effect of drivers’ demographic and socio-economic attributes, driving habits, and preferences on driver impairment, given that information on gender, age, and driving experiment is available, when the sample size increases. Therefore, the results could be generalized. Moreover, it is worth exploring the effect of road environment, traffic conditions, and the complexity of the simulated driving task on the physiological and mental workload [3,6]. To verify the findings of the current study, it is essential to measure the association between driver characteristics, music genre, and actual crash risk based on historical crash data and a naturalistic driving approach. 

## Figures and Tables

**Table 1:** The results of M-ANOVA.

Measure	Music Genre			Driver Temperament			Interaction between Music Genre and Driver Temperament		
	*F*(2, 32)	*p*	$\eta_{p}^{2}$	*F*(3, 16)	*p*	$\eta_{p}^{2}$	*F*(6, 32)	*p*	$\eta_{p}^{2}$
Mean heart rate	88.44	<0.001	0.847	7.109	0.003	0.571	20.08	<0.001	0.790
Heart rate variability	3108	<0.001	0.995	14.25	<0.001	0.728	170.2	<0.001	0.970
NASA-TLX	211.7	<0.001	0.930	10.0	0.001	0.652	29.33	<0.001	0.846
S.D. of speed	30.05	<0.001	0.653	9.495	0.001	0.640	1.444	0.229	0.213
Lane crossing frequency	47.88	<0.001	0.750	8.021	0.002	0.601	11.97	<0.001	0.692

NASA-TLX: National Aeronautics and Space Administration-Task Load Index, S.D.: standard deviation.
